# New Phenolic Derivatives of Thiazolidine-2,4-dione with Antioxidant and Antiradical Properties: Synthesis, Characterization, In Vitro Evaluation, and Quantum Studies

**DOI:** 10.3390/molecules24112060

**Published:** 2019-05-30

**Authors:** Gabriel Marc, Anca Stana, Smaranda Dafina Oniga, Adrian Pîrnău, Laurian Vlase, Ovidiu Oniga

**Affiliations:** 1 Department of Pharmaceutical Chemistry, “Iuliu Hațieganu” University of Medicine and Pharmacy, 41 Victor Babeș Street, RO-400012 Cluj-Napoca, Romania; marc.gabriel@umfcluj.ro (G.M.); onigao65@yahoo.com (O.O.); 2 Department of Therapeutic Chemistry, “Iuliu Hațieganu” University of Medicine and Pharmacy, 12 Ion Creangă Street, RO-400010 Cluj-Napoca, Romania; 3 National Institute for Research and Development of Isotopic and Molecular Technologies, RO-400293 Cluj-Napoca, Romania; adrian.pirnau@itim-cj.ro; 4 Department of Pharmaceutical Technology and Biopharmaceutics, “Iuliu Hațieganu” University of Medicine and Pharmacy, 41 Victor Babeș Street, RO-400012 Cluj-Napoca, Romania; vlaselaur@yahoo.com

**Keywords:** thiazolidine-2,4-dione, phenol, salicylamide, antioxidant, antiradical, quantum descriptors

## Abstract

Oxidative stress has been incriminated in the physiopathology of many diseases, such as diabetes, cancer, atherosclerosis, and cardiovascular and neurodegenerative diseases. There is a great interest in developing new antioxidants that could be useful for preventing and treating conditions for which oxidative stress is suggested as the root cause. The thiazolidine-2,4-dione derivatives have been reported to possess various pharmacological activities and the phenol moiety is known as a pharmacophore in many naturally occurring and synthetic antioxidants. Twelve new phenolic derivatives of thiazolidine-2,4-dione were synthesized and physicochemically characterized. The antioxidant capacity of the synthesized compounds was assessed through several in vitro antiradical, electron transfer, and Fe^2+^ chelation assays. The top polyphenolic compounds **5f** and **5l** acted as potent antiradical and electron donors, with activity comparable to the reference antioxidants used. The ferrous ion chelation capacity of the newly synthesized compounds was modest. Several quantum descriptors were calculated in order to evaluate their influence on the antioxidant and antiradical properties of the compounds and the chemoselectivity of the radical generation reactions has been evaluated. The correlation with the energetic level of the frontier orbitals partially explained the antioxidant activity, whereas a better correlation was found while evaluating the O–H bond dissociation energy of the phenolic groups.

## 1. Introduction

In the vegetable kingdom, phenols are secondary metabolites, being widely distributed in higher plants used as food, found most often in herbs and berries [1,2,3]. They have various functions in the plant, for example as ultraviolet sunscreen, signal compounds, growth regulators, and pigments [2,4].

The intense research into the field of phenolic and polyphenolic compounds performed in recent years has led to finding various pharmacological activities for this class of compounds. Some of them are linked directly to their antioxidant and antiradical potential, such as their anti-inflammatory, anti-aging, cardiovascular, and neuronal protection activity [4]. These findings are closely related to the capacity of scavenging reactive radicals. The existence of these radicals is a natural consequence of living in the aerobic environment. Many biological processes are responsible for the production of free radicals due to oxygen’s involvement in many cellular processes. Some free radicals can damage surrounding cellular structures, especially when they are produced in large amounts [3,5]. The human body has multiple enzymes to protect itself against oxidative stress, such as superoxide dismutase, catalase, and glutathione peroxidase. A supplementary contribution to these endogenous systems in fighting against oxidative stress is represented by the exogenous antioxidants taken from the diet, such as vitamins A, C, or E, and phenolic compounds [6]. Oxidative stress is involved in the physiopathology of many diseases, such as cancer, Parkinson’s, Alzheimer’s, cardiovascular and cerebrovascular diseases, alcohol-induced liver disease, and ulcerative colitis [7,8,9,10].

The development of hybrid molecules is a trend nowadays, which try to combine in the same molecule multiple pharmacophore fragments with different biological potentials [11,12,13]. The main drive of the present study was to create some thiazolidine-2,4-dione (TZD) derivatives bearing a phenolic fragment and a salicylamide moiety. The reason for choosing the TZD nucleus came from its multifarious biological potential and pharmacological activities reported in the literature. The antidiabetic activity of thiazolidine-2,4-dione derivatives was consecrated by the glitazones via activation of the PPARγ receptors [14,15,16,17]. Until now, multiple other experimental biological activities have been found for the TZD derivatives, such as the inhibition of various enzymes, such as protein tyrosine phosphatase 1B, aldose reductase, α-glucosidase, phosphoinositide-3-kinase α and γ, tyrosinase, cyclooxygenase 2, peptide deformylase, and histone deacetylase 1 [14,18]. Intense studies have been conducted to evaluate the potential anti-cancer activity of TZD derivatives, as they are considered modulators of various signaling pathways [16,18,19].

**Figure 1:**
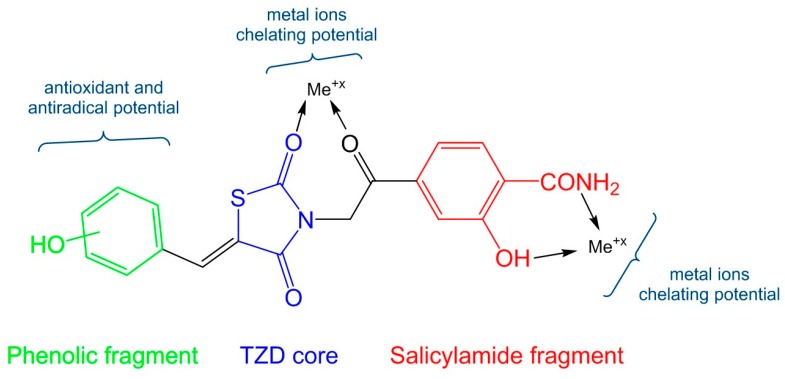
The hypothesis for designing the new compounds.

The insertion of a salicylamide fragment was performed with the scope of exploiting the potential to chelate metal ions (Figure 1). These ions are incriminated in catalyzing redox reactions that result in the formation of reactive and dangerous chemical radicals for the organism [10,20]. In addition, this fragment may improve the overall antioxidant activity of the compounds due to the presence of a phenolic OH moiety.

Research studies carried out for developing new antioxidant and antiradical compounds have indicated the existence of a relationship between these activities and the quantum properties of the molecule [21]. Therefore, a series of quantum descriptors were calculated in order to evaluate their influence on the antioxidant and antiradical properties of the compounds obtained in this study. It was reported in the literature that the number and the position of the phenolic OH groups influences the antioxidant and antiradical properties of phenolic antioxidants [22,23]. Additionally, the chemoselectivity of the radical generation reactions has been evaluated to establish how the antioxidant and antiradical properties of the synthesized compounds are influenced by their structural features, as suggested by literature data [24].

## 2. Results and Discussion

### 2.1. Chemical Synthesis

**Figure 2:**
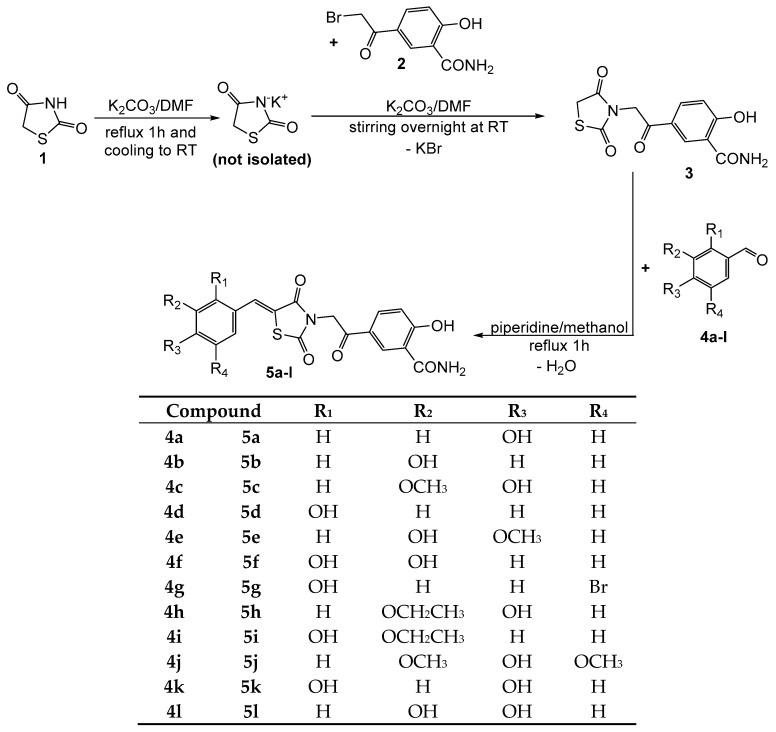
The synthetic route followed in order to obtain the final compounds **5a**–**l**.

A total of 12 new final compounds (**5a**–**l**) have been synthesized by the derivatization of the new intermediate compound **3** by Knoevenagel condensation with phenolic aromatic aldehydes (compounds **4a**–**l**) (Figure 2).

The intermediate parent (compound **3**) was obtained by N-alkylation in alkaline environment, using dimethylformamide as reaction medium, of thiazolidine-2,4-dione (compound **1**), via its potassium salt obtained in situ. The protocol used was based on some modified methods that were previously reported in the literature [25,26].

The final compounds **5a**–**l** were obtained by refluxing compound **3** with the corresponding phenolic aromatic aldehydes in methanol in order to perform the Knoevenagel condensation. In our previous research studies we reported using anhydrous sodium acetate as the catalyst and acetic acid as the solvent in order to afford the condensation between thiazolidine-2,4-dione and various phenolic aldehydes [27,28]. Unfortunately, significant changes in the amount of acetic acid used as solvent and anhydrous sodium acetate used as alkaline catalyst in the previously reported protocol have not resulted in the desired reaction products. This is possibly due to reaction conditions, most likely because of the high boiling point of acetic acid (118 °C), the polyphenolic compounds that we envisioned synthesizing decompose. TLC led to this finding, when a large number of spots were observed following development. Supplementary analyses, in order to be able to reliably identify the nature of the products that were obtained in the reaction medium, were not carried out. Therefore, we had to change the reaction conditions for conducting Knoevenagel condensation by choosing piperidine to create a basic environment, and methanol as solvent, because it has a boiling point much lower than acetic acid (64 °C vs. 118 °C) [14]. Increasing the relative amount of piperidine lowered the reaction time to 1 h, compared to the literature data that report refluxing periods from 7 to 42 h [29,30,31].

The obtained spectral data is consistent with the proposed structures. In the MS spectra of the intermediate compound **3** and the final compounds **5a**–**l** the molecular peaks were found. Analysis of the IR spectra revealed the desired signals for all compounds **3** and **5a**–**l**. The phenolic signals found were the νO–H stretching as broad bands at 3520–3554 cm^−1^ and νO–H bending bands at 1332–1370 cm^−1^. The unsubstituted amide gave the two N–H stretching bands at 3357–3423 cm^−1^ and 3164–3264 cm^−1^, respectively. Four strong νC=O stretching signals were found in the 1646–1747 cm^−1^ spectral region, two from the thiazolidine-2,4-dione ring, one from the amide, and one from the ketone group. For the methylene bridge, C–H stretching signals were found at 2926–2941 cm^−1^. In the IR spectra, the main difference between the parent compound **3** and its derivatives **5a**–**l** is the appearance of a specific signal in all final compounds **5a**–**l** of a νC=C stretching at 1609–1618 cm^−1^, proving that the Knoevenagel condensation took place successfully. In the IR spectra of the final compounds **5c**, **5e**, **5h**, **5i,** and **5j**, the presence of the etheric group was confirmed by the appearance of absorption bands corresponding to the asymmetric and symmetric stretching of the etheric bond around 1212–1244 cm^−1^ and 1027–1032 cm^−1^, respectively. For the final compound **5g**, the stretching of the C–Br bond was found at 620 cm^−1^.

In the ^1^H-NMR spectra, all the desired peaks were found, with the expected multiplicity and coupling. The protons corresponding to phenolic and amidic groups appeared as broad signals, between 9.20–14.00 ppm and 7.52–9.78 ppm, respectively. In all compounds, the protons corresponding to the methylene bridge appeared as singlets at 5.10–5.29 ppm. In the parent compound **3**, the two protons from position 5 of the thiazolidine-2,4-dione ring appeared as sharp signal at 4.40 ppm, a signal which was not found in the final compounds **5a**–**l**, proving that the Knoevenagel condensation was successful. In all final compounds **5a**–**l**, the –CH= proton from the newly introduced benzylidene moiety appeared as a sharp singlet at 7.84–8.22 ppm. This value indicates that all final compounds **5a**–**l** are in Z conformation, this being the most thermodynamically stable isomer, according to the literature [29,32,33].

In the ^13^C-NMR spectra of the synthesized compounds, the carbon from the exocyclic C=O group appeared at the highest values in the spectra, between 187.89 ppm and 189.89 ppm. Other strong de-shielded values were found for the carbons from the amide group at 170.65–171.95 ppm or for the aromatic carbons carrying the phenol groups from the salicylamide moiety at 165.36–167.69 ppm. Lower values were found for the aromatic carbons carrying the phenol groups from the benzylidene moiety, at 139.58–159.99 ppm.

### 2.2. In Vitro Antioxidant, Antiradical and Chelation Assays

The evaluation of the synthesized compounds’ antioxidant capacities was performed based on the different mechanisms possible for this activity, as reported in the literature. Compounds could manifest their activity by hydrogen atom transfer, electron transfer, or by chelation of transition metals [10]. The antioxidant potential of the synthesized thiazolidine-2,4-dione derivatives was evaluated through several in vitro assays performed at a semi-microscale level. All determinations were performed in triplicate. All assays were performed using one or more reference compounds: ascorbic acid, butylated hydroxytoluene (BHT), trolox (6-hydroxy-2,5,7,8-tetramethylchroman-2- carboxylic acid), or ethylenediamine-tetraacetic acid (EDTA) disodium salt. The obtained percentages below 10% were removed from the tables, in which the results are presented and replaced with a hyphen, meaning that the compound is considered without activity. In the same way, the values (>90%) obtained for the compounds that had exhibited a remarkable activity were marked in bold.

#### 2.2.1. Antiradical Assays

##### ABTS**·**^+^ Radical Scavenging Assay

The antioxidant activity of the tested compounds (**3** and **5a**–**l**) was expressed as percentage of the reducing of ABTS**·**^+^ radical’s initial color. Results are presented in Table 1. Some derivatives of the monophenolic aldehydes presented a modest antiradical activity (compounds **5b** and **5d**). The polyphenolic compound **5k** had a better activity, but was not significant, while the polyphenolic compounds **5f** and **5l** exhibited a much higher antioxidant activity, having a scavenging activity of 58.27% and 70.66%, respectively. Although compounds **5f**, **5k**, and **5l** have the same number of phenolic OH groups, we can observe that for the compound’s radical scavenging activity, it is important they are found in position *ortho* to each other (catechol). For the *o*-dihydroxy molecules, the hydrogen atom transfer mechanism is more favorable from the thermodynamic point of view, as suggested by previous reports. Substitution of phenols with alkyl fragments (methoxy, ethoxy) will reduce the antioxidant effect, as previously reported in the literature [13]. Compound **5l** presented the highest ABTS**·**^+^ radical scavenging activity, while the other unmentioned compounds had an ABTS**·**^+^ scavenging activity lower than 10%.

##### DPPH**·** Radical Scavenging Assay

The antioxidant potential of the tested compounds **3** and **5a**–**l** was evaluated as the potential to scavenge the DPPH**·** radicals. The greater the amount of DPPH**·** in DPPH-H, the lower the absorbance of the sample is. The obtained results are depicted in Table 1 as percentage of the reducing of DPPH**·** radical’s initial color, corresponding to the scavenging of DPPH**·**. The highest DPPH**·** scavenging activity was found for compounds **5f** and **5l** (89.61% and 92.55%, respectively). A reduced antioxidant activity compared to the top compounds was found for **5g** and **5k**, with a DPPH**·** scavenging activity equal to 12.36% and 18.13%, respectively. The other unmentioned compounds had a DPPH**·** scavenging activity lower than 10%.

The results obtained in both antiradical assays were similar, displaying compounds **5f** and **5l** as the most active phenolic thiazolidine-2,4-diones in the synthesized series of compounds, with higher radical scavenging activity than that of the reference antioxidants used in the study. These compounds are biphenolic derivatives, with the phenolic OH groups placed in *ortho* position relative to each other. Compound **5k**, another biphenolic derivative that does not have a catechol structure, has a much lower antiradical capacity than the aforementioned compounds (**5f** and **5l**). The rest of the compounds, all monophenolic derivatives, presented modest antiradical properties.

#### 2.2.2. Electron Transfer Assays

##### Ferric Reducing Antioxidant Potential (FRAP)

The tested compounds’ capacity of donating electrons was determined spectrophotometrically, using the FRAP assay. This assay is based on the reduction of ferric ions to ferrous ions by the tested compound. The resulted ferrous ions form a blue-colored complex (Fe^2+^-TPTZ) at pH = 3.6 with tripyridyltriazine (2,4,6-tris(2-pyridyl)-*s*-triazine). The amount of resulting blue complex is proportional to the capacity of the synthesized compounds to reduce the Fe^3+^ ions. The results obtained for the FRAP assay are presented in Table 2. Compounds **5f** and **5l** presented the highest electron donating potential in the synthesized series. Compound **5l** presented 91.28% of ascorbic acid’s activity.

##### Phosphomolybdate Assay for Total Antioxidant Capacity (TAC)

The assay is based on a reduction reaction of Mo^6+^ to Mo^5+^, involving the transfer mechanism of one electron at acidic pH, which results in the formation of a green phosphate Mo^5+^ complex. The higher the percent of the antioxidant power compared to a reference was, the higher the absorbance measured. The results of the TAC assay are presented in Table 2. The obtained results of the TAC assay proved a very good electron donating capacity for compounds **5f** and **5l**, similar to the reference compounds. Compound **5k** exhibited a good activity, but lower than the catechol derivatives **5f** and **5l**, displaying 51.94% of the ascorbic acid’s activity.

##### Reducing Power Assay (RP)

In this assay, the reduction of ferricyanide to ferrocyanide gave the Perl’s Prussian blue, in the presence of ferric ions. The resulted compound has an absorption peak at λ = 700 nm. The higher percent of reducing power compared to ascorbic acid, the higher the measured absorbance. The results are presented in Table 2. The results of the RP assay displayed a very good electron donating capacity for compound **5l**, similar to ascorbic acid. Compounds **5l** and **5f** surpassed BHT and trolox in terms of electron donation ability in RP assay. An intermediate activity was found for compound **5k**, which presented 46.36% of ascorbic acid’s activity.

The results of the antioxidant activity evaluation revealed that the catechol derivatives **5f** and **5l** were the most active compounds of the newly synthesized series, presenting better antioxidant potential than that of BHT and trolox, used as reference antioxidants. The rest of the synthesized compounds displayed moderate to low antioxidant capacity, inferior to that of all three antioxidant compounds used as references. It can be noticed that, regardless of the assay used, the obtained results for the antioxidant potential were similar.

#### 2.2.3. Fe^2+^ Chelation Assay

The chelating potential of the synthesized compounds was evaluated based on the potential competition for iron ions between *o*-phenanthroline and the thiazolidine-2,4-dione derivatives. A decrease in the sample absorbance indicated that the iron from the Fe^2+^-*o*-phenanthroline complex was sequestered by the tested compound. The results of the Fe^2+^ chelation assay are presented in Table 3. None of the synthesized compounds are comparable with EDTA regarding the Fe^2+^ chelating potential, as all synthesized compounds showed modest results. A very poor activity was identified for compounds **5f** and **5l**, which have a catechol group in the structure, indicating that this type of positioning of phenolic OH groups can favor Fe^2+^ chelation. Thus, we can draw a derived conclusion that the two carbonyl groups (one carbonyl group from the TZD moiety and the exocyclic ketone) or the salicylamide function do not contribute to the chelating action. This activity can only be attributed to phenolic OH groups in a relatively *ortho* position to each other, as suggested by other literature reports [10].

### 2.3. Theoretical Quantum Calculation of Chemical Descriptors

Observing the large differences regarding the antioxidant activity of the compounds **5a**–**l** obtained from the in vitro tests, we decided to perform an additional quantum study to explain the activity of these molecules in terms of quantum molecular parameters. The calculation of some significant chemical descriptors was employed in order to obtain supplementary information about the newly synthesized compounds **5a**–**l**. The requested descriptors were the three-dimensional (3D) optimized geometries and the structure of the electronic systems expressed as HOMO (highest occupied molecular orbital) and LUMO (lowest unoccupied molecular orbital), and the energy gap between those two. Table 4, Table 5 and Table 6 contain the representative quantum parameters computed for the synthesized molecules **5a**–**l**. Depiction of HOMO and LUMO of the compounds **5a**–**l** are presented in Table S1 from the Supplementary Material.

Literature reports indicated a correlation between a higher HOMO-LUMO gap of the electronic systems and a lower reactivity of the respective compounds [7,34,35]. Thus, a compound with a low HOMO-LUMO gap could be a good antioxidant. Other literature reports suggested that a good indicator of the scavenging activity of a compound is HOMO, which is linked with the electron-giving capacity of a compound and it is not directly related to the LUMO orbital energy [5,7,36,37].

The HOMO orbital is mainly located on the phenolic aromatic ring, on the TZD nucleus, or both, and less on the salicylamide-substituted ring, favoring the extraction of electrons or hydrogen atoms from it. Thus, the phenol moiety is the one mainly responsible for the antioxidant and antiradical effect of the synthesized compounds, not the salicylamide moiety. In the reactions that involve radicals (such as DPPH**·** or ABTS**·**^+^) the test compound will lose one hydrogen atom, converting itself into a radical. Therefore, based on the distribution of HOMO, we know that the hydrogen atom is extracted from the phenolic moiety and not from the salicylamide rest. The easier the radical derived from the phenolic compound can be stabilized by internal multicenter resonance, the longer its life and the lower the energy. Moreover, polyphenolic compounds, which may lose a hydrogen atom due to the presence of more phenolic OH groups in their molecule, may generate several types of radicals, depending on which phenolic OH gave up the hydrogen atom and became radicalized. It is possible to identify the chemoselectivity of the phenolic OH that is most likely to lose the hydrogen atom because the radical generated should have a lower energy, stabilizing itself by internal conjugation.

The values of E_HOMO_ found for tested compounds explain only partially the reactivity of the compounds **5a**–**l** determined in the antioxidant and antiradical assays. For compounds **5b**, **5d**, **5f**, **5g**, **5k**, and **5l** the E_HOMO_ values found were lower than 5.90 eV, chosen as an arbitrary threshold. Regarding this series of molecules, we can say that of all the synthesized compounds **5a**–**l**, they were the most active in the antiradical assays. Analyzing the antioxidant activity and the E_HOMO_ values, we can observe large discrepancies. For example, for compounds **5b**, **5d**, **5f**, and **5g**, the E_HOMO_ values were found between −6.20 eV and −6.29 eV, but the activity of compound **5f** surpasses by far the other compounds’ activities.

The second hypothesis cannot explain the relationship between the low E_HOMO_−E_LUMO_ gap values and the antioxidant action. Compounds with the lowest E_HOMO_−E_LUMO_ gap values are **5a**, **5c**, **5h,** and **5j** (under 4eV, chosen as arbitrary threshold), but these compounds exhibited a reduced activity in all the in vitro assays.

The spin density maps of the radicals produced by the compounds **5a**–**l** is depicted in Table S2 in the Supplementary Material.

Due to the failure to explain the in vitro activity of the compounds in terms of energy levels of the frontier orbitals HOMO and LUMO, we analyzed the strength of the O–H bond in the synthesized molecules, in terms of bond dissociation energy (BDE) (Table 6). The hydrogen atom transfer reaction for phenolic antioxidant compounds is linked with the O–H BDE. The weaker the O–H bond subjected to be broken is, the smaller the found BDE is, due to a lesser energy necessary to break the respective bond. In Table 6 are presented the computed O–H BDE for all phenol groups. It can be observed that for the compounds that exhibited the best activities in the in vitro assays (**5f**, **5k**, and **5l**), the BDEs are the lowest of all values presented by the newly synthesized series of compounds (71.561, 87.826, and 82.963 kcal/mol, respectively).

Based on the results obtained for the quantum chemical calculation we can conclude that the bond dissociation energy hypothesis can explain the antioxidant capacity of compounds **5a**–**l**. The substituents present in the structure of the synthesized compounds strongly influenced the antioxidant capacity, having a big impact in the breaking of the phenolic O−H bonds. By increasing the number of the phenolic groups in the molecule, the O−H bond dissociation energy will decrease, favoring the hydrogen atom transfer and improving the antioxidant activity. The relative position of the phenol units is important.

For the polyphenolic compounds, the BDE evaluation can explain which phenol is more likely to lose a hydrogen atom, thus manifesting the antioxidant activity. In the compound **5f** no significant difference was found between the bond dissociation energy of those two O−H bonds (71.561 kcal/mol in *ortho* vs. 71.668 kcal/mol in *meta*). In compounds **5k** and **5l** the phenol from *para* is more likely to break (87.826 kcal/mol and 82.963 kcal/mol, respectively), instead of the phenol from position *ortho* in compound **5k** (89.684 kcal/mol) and **5l** (95.971 kcal/mol). Analyzing this data we can conclude that the position of the phenols on the aromatic ring is important for this activity, as presented in the literature [3].

## 3. Materials and Methods

### 3.1. Chemistry

All chemicals used for the synthesis, purification, analysis, and antioxidant assays, with appropriate grade purity, were purchased from local suppliers and were used as supplied. The melting points were measured using an MPM-H1 melting point device (Schorpp Gerätetechnik, Überlingen, Germany), based on the glass capillary method. The MS spectra of the compounds were recorded using an Agilent 1100 series device in positive ionization mode for intermediate compound **3** and in negative ionization mode for the final compounds **5a**–**l**, connected to an Agilent Ion Trap SL mass spectrometer (70 eV) instrument (Agilent Technologies, Santa Clara, CA, USA). The IR spectra were recorded under vacuum, using a FT/IR 6100 spectrometer (Jasco, Cremella, Italy) in KBr pellets. The ^1^H-NMR and ^13^C-NMR spectra were recorded using an Avance NMR spectrometer (Bruker, Karlsruhe, Germany) in dimethyl sulfoxide-*d*_6_. Chemical shift values were reported in *δ* units, relative to tetramethylsilane as internal standard.

#### 3.1.1. Synthesis of Compound **3**

In a glass flask, 15 mL dimethylformamide (DMF) were added to 10 mmol (1.17 g) of thiazolidin-2,4-dione (compound **1**) and 10 mmol (1.38 g) of anhydrous potassium carbonate. The mixture was refluxed gently under condenser for one hour, in order to obtain the potassium salt of thiazolidin-2,4-dione in situ (Figure 2). The glass flask was left to stand at room temperature to cool down. To the obtained reaction mixture, another 10 mmol (1.38 g) of anhydrous potassium carbonate were added in order to ensure an alkaline environment during the next reaction. The entire amount of potassium carbonate was not added from the beginning because some degradation was observed at reflux in the presence of a greater amount than the required stoichiometry of potassium carbonate. Using a magnetic stirrer, the suspension was vigorously mixed, while adding 10 mmol (2.57 g) of 5-(2-bromoacetyl)-2-hydroxy-benzamide (compound **2**). After a few moments, an abundant precipitate appeared. The content of the reaction flask was mixed overnight. After the reaction’s completion was confirmed by TLC, the reaction mixture was poured into ice cold brine. Then, a 10% sulfuric acid solution was added dropwise until total precipitation of the product. The resulted precipitate was filtered, washed with fresh water, and dried under vacuum. The impure solid was recrystallized from a mixture of ethanol:DMF, giving the pure product as a white solid.

*5-(2-(2,4-dioxothiazolidin-3-yl)acetyl)-2-hydroxybenzamide* (**3**): white solid; mp = 222 °C; yield = 43–51%; FT IR (KBr) ν_max_ cm^−1^: 3520 (str O–H), 3370, 3181 (N–H amide), 2933 (CH_2_), 1747, 1689, 1674, 1657 (str C=O), 1332 (bend OH); MS: *m*/*z* = 294.9 (M + 1); ^1^H-NMR (DMSO-*d*_6_, 500 MHz) δ: 13.95 (br, 1H, OH), 8.72 (br, 1H, NH), 8.63 (s, 1H, Ar), 8.17 (br, 1H, NH), 8.06 (d, 1H, Ar), 7.05 (d, 1H, Ar), 5.10 (s, 2H, –CH_2_–), 4.40 (s, 2H, –CH_2_–); ^13^C-NMR (DMSO-*d*_6_, 125 MHz) δ: 189.61 (C=O), 177.45 (C=O), 172.15 (C=O), 171.94 (C=O), 166.52 (ArC–O), 134.20, 130.44, 125.40, 118.73, 114.64 (5 aromatic carbons), 47.70 (–CH_2_–), 34.58 (–CH_2_–).

#### 3.1.2. Synthesis of Compounds **5a**–**l**

In a glass flask, 2 mmol (0.588 g) of compound **3** and 2 mmol of the appropriate aldehyde (compounds **4a**–**l**) were mixed with 5 mL of methanol. Later, 4 mmol (0.32 g) of piperidine were added dropwise and the mixture was gently refluxed under condenser for one hour. The reaction mixture was then left to stay overnight at room temperature in order to remove by evaporation some methanol to get a more concentrated solution. The solution was mixed in a glass flask with ice and water, and a 10% hydrochloric acid solution was added dropwise until total precipitation of the desired product. The flask was left in a refrigerator for a few hours to favor the quantitative precipitation of the product. The precipitate was filtered under vacuum and crystalized twice from diethyl ether to get the pure final compounds.

*(Z)-2-hydroxy-5-(2-(5-(4-hydroxybenzylidene)-2,4-dioxothiazolidin-3-yl)acetyl) benzamide* (**5a**): intense yellow solid; carbonization over 260 °C; yield = 56%; FT IR (KBr) ν_max_ cm^−1^: 3546 (str O–H), 3419, 3179 (N–H amide), 2930 (CH_2_), 1738, 1686, 1671, 1655 (str C=O), 1614 (C=C), 1357 (bend OH); MS: *m*/*z* = 397.2 (M − 1); ^1^H-NMR (DMSO-*d*_6_, 500 MHz) δ: 13.21 (br, 1H, OH), 10.36 (br, 1H, OH), 8.69 (s, 1H, Ar), 8.59 (br, 1H, NH), 8.09 (d, 1H, Ar), 8.01 (br, 1H, NH), 7.87 (s, 1H, –CH=), 7.58 (d, 2H, Ar), 7.05 (d, 1H, Ar), 6.89 (d, 2H, Ar), 5.19 (s, 2H, –CH_2_–); ^13^C-NMR (DMSO-*d*_6_, 125 MHz) δ: 187.99 (C=O), 170.82 (C=O), 167.26 (C=O), 166.84 (C=O), 166.80 (ArC–O), 159.99 (ArC–O), 134.62 (–CH=), 133.41, 133.06, 125.60, 129.96, 125.37, 118.92, 117.02, 114.57 (8 aromatic carbons), 116.51 (TZD C_5_), 45.93 (–CH_2_–).

*(Z)-2-hydroxy-5-(2-(5-(3-hydroxybenzylidene)-2,4-dioxothiazolidin-3-yl)acetyl)benzamide* (**5b**): yellow solid; carbonization over 260 °C; yield = 40%; FT IR (KBr) ν_max_ cm^−1^: 3550 (str O–H), 3381, 3208 (N–H amide), 2928 (CH_2_), 1747, 1694, 1680, 1658 (str C=O), 1613 (C=C), 1357 (bend OH); MS: *m*/*z* = 397.2 (M − 1); ^1^H-NMR (DMSO-*d*_6_, 500 MHz) δ: 13.98 (br, 1H, OH), 9.90 (br, 1H, OH), 8.75 (br, 1H, NH), 8.67 (s, 1H, Ar), 8.19 (br, 1H, NH), 8.08 (d, 1H, Ar), 7.92 (s, 1H, –CH=), 7.38 (t, 1H, Ar), 7.16 (d, 1H, Ar), 7.07–7.06 (m, 2H, Ar), 6.94 (d, 1H, Ar), 5.29 (s, 2H, –CH_2_–); ^13^C-NMR (DMSO-*d*_6_, 125 MHz) δ: 189.57 (C=O), 171.95 (C=O), 167.71 (C=O), 166.64 (C=O), 165.78 (ArC–O), 158.45 (ArC–O), 134.50, 134.29, 130.56, 125.29, 121.99, 121.20, 118.88, 118.68, 117.78, 114.67 (10 aromatic carbons), 131.00 (–CH=), 116.61 (TZD C_5_), 44.08 (–CH_2_–).

*(Z)-2-hydroxy-5-(2-(5-(4-hydroxy-3-methoxybenzylidene)-2,4-dioxothiazolidin-3-yl)acetyl)benzamide* (**5c**): yellow solid; carbonization over 280 °C; yield = 31%; FT IR (KBr) ν_max_ cm^−1^: 3545 (str O–H), 3374, 3234 (N–H amide), 2929 (CH_2_), 1745, 1680, 1663, 1655 (str C=O), 1616 (C=C), 1357 (bend OH), 1212, 1031 (str C–O–C asymm and symm); MS: *m*/*z* = 272.2 (M − 1); ^1^H-NMR (DMSO-*d*_6_, 500 MHz) δ: 13.01 (br, 1H, OH), 10.29 (br, 1H, OH), 9.53 (br, 1H, NH), 8.71 (s, 1H, Ar), 8.66 (br, 1H, NH), 7.99 (d, 1H, Ar), 7.89 (d, 1H, –CH=), 7.61–7.59 (m, 2H, Ar), 7.01 (d, 1H, Ar), 6.95 (d, 1H, Ar), 5.18 (s, 2H, –CH_2_–), 3.71 (s, 3H, –CH_3_); ^13^C-NMR (DMSO-*d*_6_, 125 MHz) δ: 188.16 (C=O), 171.24 (C=O), 166.82 (C=O), 166.35 (C=O), 166.11 (ArC–O), 148.16 (ArC–O), 147.51 (ArC–O), 135.11, 130.51, 120.14, 126.94, 125.41, 119.47, 116.10, 115.29, 113.26 (9 aromatic carbons), 133.57 (–CH=), 116.64 (TZD C_5_), 57.43 (–CH_3_), 46.80 (–CH_2_–).

*(Z)-2-hydroxy-5-(2-(5-(2-hydroxybenzylidene)-2,4-dioxothiazolidin-3-yl)acetyl)benzamide* (**5d**): orange solid; mp = 225 °C; yield = 39%; FT IR (KBr) ν_max_ cm^−1^: 3554 (str O–H), 3386, 3219 (N–H amide), 2928 (CH_2_), 1736, 1680, 1658, 1647 (str C=O), 1617 (C=C), 1359 (bend OH); MS: *m*/*z* = 397.1 (M − 1); ^1^H-NMR (DMSO-*d*_6_, 500 MHz) δ: 12.98 (br, 1H, OH), 10.57 (br, 1H, OH), 8.91 (br, 1H, NH), 8.59 (s, 1H, Ar), 8.22 (br, 1H, NH), 8.18 (s, 1H, –CH=), 8.01 (d, 1H, Ar), 7.35–7.38 (m, 2H, Ar), 7.04 (d, 1H, Ar), 6.98–6.96 (m, 2H, Ar), 5.14 (s, 2H, –CH_2_–); ^13^C-NMR (DMSO-*d*_6_, 125 MHz) δ: 188.27 (C=O), 171.55 (C=O), 167.44 (C=O), 167.29 (ArC–O), 166.68 (C=O), 157.39 (ArC–O), 134.55, 130.01, 129.25, 129.01, 125.37, 121.52, 121.14, 120.96, 120.28, 116.23 (10 aromatic carbons), 132.87 (–CH=), 116.91 (TZD C_5_), 44.37 (–CH_2_–).

*(Z)-2-hydroxy-5-(2-(5-(3-hydroxy-4-methoxybenzylidene)-2,4-dioxothiazolidin-3-yl)acetyl)benzamide* (**5e)**: pale yellow solid; mp = 216 °C; yield = 46%; FT IR (KBr) ν_max_ cm^−1^: 3546 (str O–H), 3359, 3192 (N–H amide), 2935 (CH_2_), 1742, 1693, 1678, 1655 (str C=O), 1617 (C=C), 1334 (bend OH), 1243, 1029 (str C–O–C asymm and symm); MS: *m*/*z* = 427.4 (M − 1); ^1^H-NMR (DMSO-*d*_6_, 500 MHz) δ: 13.14 (br, 1H, OH), 9.20 (br, 1H, OH), 8.99 (br, 1H, NH), 8.63 (s, 1H, Ar), 8.50 (br, 1H, NH), 7.96 (d, 1H, Ar), 7.91 (s, 1H, –CH=), 7.36 (d, 1H, Ar), 7.29 (s, 1H, Ar), 7.04 (d, 1H, Ar), 6.99 (d, 1H, Ar), 5.23 (s, 2H, –CH2–), 3.79 (s, 3H, –CH_3_); ^13^C-NMR (DMSO-*d*_6_, 125 MHz) δ: 188.91 (C=O), 170.89 (C=O), 166.22 (C=O), 165.61 (C=O), 165.36 (ArC–O), 146.89 (ArC–O), 146.19 (ArC–O), 135.09, 132.41, 130.26, 125.37, 120.64, 118.47, 116.76, 114.52, 112.96 (9 aromatic carbons), 132.12 (–CH=), 116.82 (TZD C_5_), 58.11 (–CH_3_), 45.56 (–CH2–).

*(Z)-5-(2-(5-(2,3-dihydroxybenzylidene)-2,4-dioxothiazolidin-3-yl)acetyl)-2-hydroxy benzamide* (**5f**): yellow solid; mp = 254 °C; yield = 61%; FT IR (KBr) ν_max_ cm^−1^: 3540 (str O–H), 3365, 3164 (N–H amide), 2939 (CH_2_), 1733, 1678, 1671, 1648 (str C=O), 1609 (C=C), 1358 (bend OH); MS: *m*/*z* = 413.1 (M − 1); ^1^H-NMR (DMSO-*d*_6_, 500 MHz) δ: 12.84 (br, 1H, OH), 9.95 (br, 2H, OH), 8.95 (br, 1H, NH), 8.59 (s, 1H, Ar), 8.45 (br, 1H, NH), 8.16 (s, 1H, –CH=), 8.02 (d, 1H, Ar), 7.28 (d, 1H, Ar), 7.04 (d, 1H, Ar), 6.71–6.73 (m, 2H, Ar), 5.16 (s, 2H, –CH2–); ^13^C-NMR (DMSO-*d*_6_, 125 MHz) δ: 189.16 (C=O), 170.98 (C=O), 166.87 (C=O), 165.89 (C=O), 165.38 (ArC–O), 150.23 (ArC–O), 146.19 (ArC–O), 133.98, 129.91, 125.39, 121.87, 121.80, 117.09, 117.01, 116.82, 116.70 (9 aromatic carbons), 132.81 (–CH=), 116.54 (TZD C_5_), 47.49 (–CH2–).

*(Z)-5-(2-(5-(5-bromo-2-hydroxybenzylidene)-2,4-dioxothiazolidin-3-yl)acetyl)-2-hydroxybenzamide* (**5g**): yellow solid; mp = 235 °C; yield = 76%; FT IR (KBr) ν_max_ cm^−1^: 3543 (str O–H), 3419, 3192 (N–H amide), 2926 (CH_2_), 1729, 1670, 1660, 1648 (str C=O), 1615 (C=C), 1356 (bend OH), 620 (str C-Br); MS: *m*/*z* = 475.7 (M − 1) with bromine isotopic pattern; ^1^H-NMR (DMSO-*d*_6_, 500 MHz) δ: 12.71 (br, 1H, OH), 10.59 (br, 1H, OH), 9.43 (br, 1H, NH), 9.01 (br, 1H, NH), 8.66 (s, 1H, Ar), 8.10–8.09 (m, 2H, Ar, –CH=), 7.90 (s, 1H, Ar), 7.14 (d, 1H, Ar), 7.04 (d, 1H, Ar), 6.90 (d, 1H, Ar), 5.19 (s, 2H, Ar); ^13^C-NMR (DMSO-*d*_6_, 125 MHz) δ: 188.61 (C=O), 171.09 (C=O), 167.41 (ArC–O), 166.80 (C=O), 166.17 (C=O), 157.87 (ArC–O), 134.26, 130.99, 130.29, 129.83, 125.37, 121.63, 119.54, 118.99, 118.02, 115.22 (10 aromatic carbons), 132.96 (–CH=), 116.73 (TZD C_5_), 46.49 (–CH2–).

*(Z)-5-(2-(5-(3-ethoxy-4-hydroxybenzylidene)-2,4-dioxothiazolidin-3-yl)acetyl)-2-hydroxybenzamide* (**5h**): yellow solid; carbonization over 290 °C; yield = 65%; FT IR (KBr) ν_max_ cm^−1^: 3547 (str O–H), 3418, 3237 (N–H amide), 2928 (CH_2_), 1740, 1680, 1671, 1661 (str C=O), 1615 (C=C), 1360 (bend OH), 1244, 1032 (str C–O–C asymm and symm); MS: *m*/*z* = 441.2 (M − 1); ^1^H-NMR (DMSO-*d*_6_, 500 MHz) δ: 12.54 (br, 1H, OH), 9.96 (br, 1H, OH), 9.01 (br, 1H, NH), 8.64 (s, 1H, Ar), 8.50 (br, 1H, NH), 7.94 (d, 1H, Ar), 7.85 (s, 1H, –CH=), 7.38–7.35 (m, 2H, Ar), 7.05 (d, 1H, Ar), 6.94 (d, 1H, Ar), 5.22 (s, 2H, –CH2–), 4.10 (q, 2H, –CH2–), 1.53 (t, 3H, –CH_3_); ^13^C-NMR (DMSO-*d*_6_, 125 MHz) δ: 188.53 (C=O), 171.24 (C=O), 167.26 (C=O), 166.84 (C=O), 165.43 (ArC–O), 149.51 (ArC–O), 146.01 (ArC–O), 134.43, 129.86, 129.47, 125.30, 123.42, 120.73, 116.21, 115.92, 114.47 (9 aromatic carbons), 133.84 (–CH=), 116.62 (TZD C_5_), 65.01 (–CH2–), 44.55 (–CH2–), 16.26 (–CH_3_).

*(Z)-5-(2-(5-(3-ethoxy-2-hydroxybenzylidene)-2,4-dioxothiazolidin-3-yl)acetyl)-2-hydroxybenzamide* (**5i**): yellow solid; mp = 227°C; yield=71%; FT IR (KBr) ν_max_ cm^−1^: 3545 (str O–H), 3417, 3241 (N–H amide), 2941 (CH_2_), 1738, 1678, 1656, 1649 (str C=O), 1615 (C=C), 1354 (bend OH), 1236, 1027 (str C–O–C asymm and symm); MS: *m*/*z* = 441.2 (M − 1); ^1^H-NMR (DMSO-*d*_6_, 500 MHz) δ: 12.66 (br, 1H, OH), 10.26 (br, 1H, OH), 9.66 (br, 1H, NH), 8.57 (s, 1H, Ar), 8.22 (s, 1H, –CH=), 7.86 (d, 1H, Ar), 7.52 (br, 1H, NH), 7.03 (d, 1H, Ar), 7.12 (d, 1H, Ar), 6.94 (t, 1H, Ar), 6.70 (d, 1H, Ar), 5.14 (s, 2H, –CH2–), 4.12 (q, 2H, –CH2–), 1.38 (t, 3H, –CH_3_); ^13^C-NMR (DMSO-*d*_6_, 125 MHz) δ: 187.89 (C=O), 170.65 (C=O), 167.90 (C=O), 167.13 (ArC–O), 166.07 (C=O), 147.77 (ArC–O), 147.73 (ArC–O), 133.12, 132.04, 129.46, 125.29, 120.67, 120.27, 120.15, 116.88, 115.88 (9 aromatic carbons), 132.01 (–CH=), 120.89 (TZD C_5_), 64.85 (–CH2–), 47.55 (–CH2–), 15.05 (–CH_3_).

*(Z)-2-hydroxy-5-(2-(5-(4-hydroxy-3,5-dimethoxybenzylidene)-2,4-dioxothiazolidin-3-yl)acetyl)benzamide* (**5j**): yellow solid; mp = 302 °C; yield = 64%; FT IR (KBr) ν_max_ cm^−1^: 3546 (str O–H), 3423, 3203 (N–H amide), 2932 (CH_2_), 1743, 1682, 1667, 1648 (str C=O), 1617 (C=C), 1360 (bend OH), 1243, 1027 (str C–O–C asymm and symm); MS: *m*/*z* = 457.3 (M − 1); ^1^H-NMR (DMSO-*d*_6_, 500 MHz) δ: 14.00 (br, 1H, OH), 9.44 (br, 1H, OH), 8.81 (br, 1H, NH), 8.75 (br, 1H, NH), 8.71 (s, 1H, Ar), 8.07 (d, 1H, Ar), 7.93 (s, 1H, –CH=), 7.07 (d, 1H, Ar), 6.99 (s, 2H, Ar), 5.29 (s, 2H, –CH2–), 3.85 (s, 6H, –CH_3_); ^13^C-NMR (DMSO-*d*_6_, 125 MHz) δ: 189.69 (C=O), 171.91 (C=O), 167.69 (ArC–O), 166.59 (C=O), 165.84 (C=O), 148.78 (ArC–O), 139.58 (ArC–O), 135.27, 130.60, 125.34, 123.56, 118.76, 114.70, 108.80 (7 aromatic carbons), 134.24 (–CH=), 117.42 (TZD C_5_), 56.62 (–CH_3_), 48.08 (–CH2–).

*(Z)-5-(2-(5-(2,4-dihydroxybenzylidene)-2,4-dioxothiazolidin-3-yl)acetyl)-2-hydroxybenzamide* (**5k**): yellow mustard solid; carbonization over 250 °C; yield = 68%; FT IR (KBr) ν_max_ cm^−1^: 3544 (str O–H), 3420, 3220 (N–H amide), 2938 (CH_2_), 1729, 1680, 1661, 1647 (str C=O), 1618 (C=C), 1370 (bend OH); MS: *m*/*z* = 413.0 (M − 1); ^1^H-NMR (DMSO-*d*_6_, 500 MHz) δ: 13.53 (br, 1H, OH), 10.40 (br, 2H, OH), 8.83 (br, 1H, NH), 8.68 (s, 1H, Ar), 8.42 (br, 1H, NH), 8.14 (s, 1H, –CH=), 7.99 (d, 1H, Ar), 7.54 (d, 1H, Ar), 7.04 (d, 1H, Ar), 6.65 (d, 1H, Ar), 6.34 (s, 1H, Ar), 5.19 (s, 2H, –CH2–); ^13^C-NMR (DMSO-*d*_6_, 125 MHz) δ: 188.73 (C=O), 171.50 (C=O), 166.65 (C=O), 166.21 (ArC–O), 165.82 (C=O), 161.66 (ArC–O), 156.81 (ArC–O), 135.15, 131.14, 130.22, 125.31, 121.47, 119.84, 116.19, 111.82, 104.15 (9 aromatic carbons), 133.31 (–CH=), 116.80 (TZD C_5_), 47.56 (–CH2–).

*(Z)-5-(2-(5-(3,4-dihydroxybenzylidene)-2,4-dioxothiazolidin-3-yl)acetyl)-2-hydroxybenzamide* (**5l**): dark orange solid; carbonization over 260 °C; yield = 76%; FT IR (KBr) ν_max_ cm^−1^: 3543 (str O–H), 3357, 3264 (N–H amide), 2936 (CH_2_), 1736, 1679, 1662, 1646 (str C=O), 1618 (C=C), 1363 (bend OH); MS: *m*/*z* = 413.0 (M − 1); ^1^H-NMR (DMSO-*d*_6_, 500 MHz) δ: 13.41 (br, 1H, OH), 9.81 (br, 2H, OH), 9.78 (br, 1H, NH), 9.24 (br, 1H, NH), 8.67 (s, 1H, Ar), 8.06 (d, 1H, Ar), 7.84 (s, 1H, –CH=), 7.21 (d, 1H, Ar), 7.05–7.03 (m, 2H, Ar), 6.85 (d, 1H, Ar), 5.17 (s, 2H, –CH2–); ^13^C-NMR (DMSO-*d*_6_, 125 MHz) δ: 187.93 (C=O), 170.99 (C=O), 166.89 (C=O), 166.60 (C=O), 166.26 (ArC–O), 146.16 (ArC–O), 146.98 (ArC–O), 133.84, 130.05, 128.55, 127.84, 125.39, 120.56, 116.52, 115.29, 114.29 (9 aromatic carbons), 133.61 (–CH=), 116.90 (TZD C_5_), 45.99 (–CH2–).

### 3.2. In Vitro Antioxidant, Antiradical and Chelation Assays

The stock solutions of the tested compounds (**3**, **5a**–**l**) have been prepared by dissolving the solid powders in DMSO, with resulting concentrations of 1 mg/mL. The spectrophotometrically in vitro assays were performed in cuvettes of poly(methyl methacrylate) (PMMA) with 10 mm width, using an UV-VIS spectrophotometer Jasco V-530 (Jasco International Co., Tokyo, Japan).

The absorption spectra of the compounds in the region visible spectrum between 430 nm and 700 nm indicated that none of the tested compounds have absorption peaks near the wavelengths where the antioxidant and antiradical assays were performed (510 nm, 517 nm, 593 nm, 695 nm, and 700 nm). All the assays were performed in triplicate, mean values of three different measurements were reported.

#### 3.2.1. Antiradical assays

##### ABTS**·**^+^ radical scavenging assay

The ABTS**·**^+^ (2,2′-azinobis-(3-ethylbenzothiazoline-6-sulfonic acid) decolorization assay to ABTS in the presence of a hydrogen-donating oxidant was based on the principle reported by Re et al., which suffered adaptation to a semi-microscale assay [20,38]. ABTS**·**^+^ cationic radical was prepared by dissolving 0.5 g of solid ABTS, and as a radical generator, 0.7 g of MnO_2_ in 100 mL of potassium phosphate buffer (0.1 M, pH = 7.4) [9]. The solution was kept closed in the dark at room temperature overnight to generate the green ABTS**·**^+^ cationic radicals. The solution was filtered and the resulted solution’s absorbance was adjusted at approximately 0.7 by progressively adding potassium phosphate buffer (0.1 M, pH = 7.4) to create the working stock solution of the ABTS**·**^+^ monocationic radical. Prior to usage, the reagent solutions’ absorbance stability was verified at λ = 734 nm for one hour to ensure the constant absorption of the reagent and its stability. To 30 µL of test samples solutions and trolox used as control, 2 mL of ABTS**·**^+^ reagent were added and mixed thoroughly. The mixture was shaken well over 10 min at room temperature. The absorbance of the resulted solutions were determined spectrophotometrically at λ = 734 nm against a blank sample used as reference. The activity of the tested compounds was assessed using formula [39]:

$$\%\;\mathrm{ABTS}\;\mathrm{scavenging}\;=\;\frac{\mathrm{control}\;\mathrm{absorbance}-\mathrm{sample}\;\mathrm{absorbance}}{\mathrm{control}\;\mathrm{absorbance}}\times 100$$

##### DPPH**·** Radical Scavenging Assay

The DPPH**·** radical scavenging assay was performed by an adaptation of some reported protocols from the literature [9,40,41], based on the initial report of Brand-Williams et al. [42]. The assay is based on the transfer of one proton from the analyzed substrate to the stable free radical of DPPH**·** (2,2-diphenyl-1-picrylhydrazyl). This proton transfer will turn the violet DPPH**·** radical to a light yellow compound. The loss of the intense violet color is proportional to the amount of DPPH**·** radical converted.

The initial solution of DPPH**·** was obtained by dissolving 20 mg of DPPH**·** in 200 mL of methanol. Later, the working DPPH**·** solution was obtained by diluting with methanol the initial solution to an absorbance value of approximately 1 at λ = 517 nm. Over 40 µL of the test samples and controls solutions, 2 mL solution of DPPH in methanol were added. The mixture was shaken from time to time over 30 min at room temperature in the absence of light. The absorbance of the resulted solutions were determined spectrophotometrically at λ = 517 nm against a blank sample. The percent of DPPH radical scavenging activity of tested compounds was assessed using formula [39]:

$$\%\;\mathrm{DPPH}\;\mathrm{scavenging}\;=\;\frac{\mathrm{control}\;\mathrm{absorbance}-\mathrm{sample}\;\mathrm{absorbance}}{\mathrm{control}\;\mathrm{absorbance}}\times 100$$

#### 3.2.2. Electron Transfer assays

##### Ferric Reducing Antioxidant Potential (FRAP)

The reducing power of the tested compounds was determined using the FRAP assay, according to a modified method proposed initially by Benzie and Strain [9,41,43,44]. For this 50 μL solution of each compound were mixed with 1000 μL FRAP reagent [9,43] and the resulted mixtures were shaken vigorously for 30 min. Their absorbance was measured at λ = 593 nm against a blank sample prepared from 50 μL DMSO and 1000 μL FRAP reagent. The reducing power of each compound was expressed as percent of the most active reference compound in the current assay, based on the formula:

$$\%\;\mathrm{of}\;\mathrm{control}\;\mathrm{ferric}\;\mathrm{reducing}\;\mathrm{power}=\;\frac{\mathrm{sample}\;\mathrm{absorbance}}{\mathrm{reference}\;\mathrm{absorbance}}\times 100$$

##### Phosphomolybdate Assay for Total Antioxidant Capacity (TAC)

To determine the TAC of the tested compounds we used a procedure previously reported in the literature [9,45,46], with slight modifications. For this 100 μL of each compound’s solution (1 mg/mL in DMSO) were mixed with 1 mL reagent [9] in test tubes, mixed well, and incubated for 90 min in a water bath at 95 °C. After cooling at room temperature, the absorbance of the samples was measured against a blank sample at λ = 695 nm. The reducing power of each compound was expressed as percent of the most active reference compound in the current assay, based on the formula:

$$\%\;\mathrm{of}\;\mathrm{control}\;\mathrm{ferric}\;\mathrm{reducing}\;\mathrm{power}=\;\frac{\mathrm{sample}\;\mathrm{absorbance}}{\mathrm{reference}\;\mathrm{absorbance}}\times 100$$

##### Reducing Power Assay (RP)

The principle driving this method is based on increasing the absorbance in the final test tubes, in correlation with the antioxidant activity. In this assay the tested compound reduces ferric ion from potassium ferricyanide and in the presence of ferric ions, the resulted ferrocyanide gives a blue complex. The current assay was adapted to a semi-microscale based on previous literature reports [9,47]. In glass test tubes, 0.1 mL of the samples solutions were mixed with 1 mL DMSO, 0.4 mL phosphate buffer (0.2 M, pH = 6.6), and 0.4 mL K_3_[Fe(CN)_6_] solution (1% *w*/*v*). The mixture was incubated in a water bath at 50 °C for 20 min. After cooling at room temperature, 0.5 mL trichloroacetic acid (10% *w*/*w*) was added. The resulting mixture was left to stand for 30 min, with resulting precipitates eventually depositing on the bottom of the test tubes. Then, 0.25 mL of the solution was collected carefully and mixed with 0.14 mL FeCl_3_ solution (0.1% *w*/*v*) and 0.75 mL distilled water. The absorbance was measured at λ = 700 nm against a blank sample. The reducing power of each compound was expressed as percent of the most active reference compound in the current assay, based on the formula:

$$\%\;\mathrm{of}\;\mathrm{control}\;\mathrm{ferric}\;\mathrm{reducing}\;\mathrm{power}=\;\frac{\mathrm{sample}\;\mathrm{absorbance}}{\mathrm{reference}\;\mathrm{absorbance}}\times 100$$

#### 3.2.3. Fe^2+^ Chelation Assay

The protocol used for evaluating Fe^2+^ chelating ability of the compounds was adapted from the initial report of Benzie and Strain [43,48,49]. The assay is based on the formation of a colored complex between Fe^2+^ and *o*-phenanthroline, which could be disrupted by the presence of a chelating compound with a higher affinity for the ferrous ions. For this 0.5 mL of sample solution of the tested compounds was mixed with 0.25 mL of *o*-phenanthroline solution 0.05% in methanol and 0.5 mL FeCl_3_ solution 200 µM. The obtained solutions were left to rest for 10 min at room temperature, and then the solutions’ absorbances were measured at λ = 510 nm against a blank sample. The results were calculated using the formula:

$$\mathrm{iron}\;\mathrm{chelation}\;\mathrm{capacity}\;\left(\%\right)\;=\;\frac{\mathrm{control}\;\mathrm{absorbance}-\mathrm{sample}\;\mathrm{absorbance}}{\mathrm{control}\;\mathrm{absorbance}}\times 100$$

### 3.3. Theoretical Quantum Calculation of Chemical Descriptors

In order to evaluate the correlation between the antioxidant and the antiradical activity of the studied compounds **5a**–**l**, a quantum chemical study was carried out. The frontier molecular orbitals (E_HOMO_ and E_LUMO_), enthalpies of molecules (H), and some derived descriptors were computed using the Density Functional Theory (DFT) on the basis of hybrid B3LYP potential with 6-31G* basis set, as reported previously in the literature [44]. Literature reports indicate that a close connection could be made between the practical assay and the theoretical properties via calculation of molecular properties with DFT [37].

We have evaluated in silico the potential antiradical effect as a consequence of the hydrogen atom transfer mechanism, which is more favorable for phenolic compounds, especially for *ortho*-dihydroxy compounds [13]. The bond dissociation energy (BDE) is influenced by the parent molecule’s stability and that of the corresponding phenoxyl radical. The BDE of O–H values were computed as the enthalpy difference at 298 K for the homolytic reaction: Ar–OH → Ar–O**˙** + **˙**H, where Ar–O**˙** is the corresponding phenoxyl radical of the parent phenolic compound. Lower BDE values are characteristic for compounds with better antioxidant properties [5]. The calculation of the BDE was assessed using the formula: BDE (O–H) = H (Ar–O**˙**) + H (**˙**H) – H (Ar–OH), where H (**˙**H) = −0.498 Hartrees = −312.956 kcal/mol [4,13].

## 4. Conclusions

Twelve new phenolic derivatives of thiazolidine-2,4-dione were synthesized. As a result of antioxidant and antiradical studies, we found that in most cases, these activities are linked to the number of phenolic OH groups present in the molecules. In the case of bi-phenolic compounds, which have the two OH groups on different aromatic nuclei, we can notice a modest activity. In the case of tri-phenolic compounds, those having an OH group on an aromatic ring (from the salicylamide rest) and the other two phenolic groups on the other aromatic nucleus exhibited a much better activity (compounds **5f**, **5k**, and **5l**). Of these compounds, the catechol derivatives **5f** and **5l** presented a similar or higher activity than the antioxidant standards used. This suggested that the positioning of the OH groups in the adjacent position (catechol) greatly increases the antioxidant and antiradical activity, in comparison to the compounds, in which they are oriented in the meta position relative to each other (compound **5k**). The importance of the catechol group for a better antioxidant activity was reported in previous studies in the literature [24]. The top polyphenolic compounds **5f** and **5l** act as potent antiradical and electron donors, with activity comparable to the antioxidants used as reference compounds. The substitution of OH with O-alkyl is ineffective, greatly reducing the antioxidant action. The ferrous ion chelation capacity of the newly synthesized compounds is negligible.

The correlation with the energetic level of the frontier orbitals explains only partially the antioxidant activity. Better correlation was found while evaluating the O–H bond dissociation energy of the phenolic groups.

## Figures and Tables

**Table 1:** Results of the ABTS**·**^+^ and DPPH**·** scavenging assays.

Compound	% of Radical Scavenging	
	ABTS·^+^	DPPH·
**3**	-	-
**5a**	-	-
**5b**	12.11	-
**5c**	-	-
**5d**	15.81	-
**5e**	-	-
**5f**	58.27	89.61
**5g**	-	12.36
**5h**	-	-
**5i**	-	-
**5j**	-	-
**5k**	22.75	18.13
**5l**	70.66	**92.55**
Ascorbic acid	N.T.	77.20
BHT	N.T.	63.50
Trolox	54.35	73.62

N.T. = not tested; BHT = butylated hydroxytoluene. The values obtained for the most active compounds (>90%) are marked in bold.

**Table 2:** Results of the Ferric Reducing Antioxidant Potential (FRAP), Total Antioxidant Capacity (TAC), and Reducing Power (RP) Assays, expressed as % activity of the most active control (ascorbic acid).

Compound	FRAP	TAC	RP
**3**	11.86	-	-
**5a**	-	-	14.75
**5b**	-	-	23.82
**5c**	-	23.05	15.20
**5d**	23.69	17.61	16.54
**5e**	16.26	-	18.32
**5f**	86.12	**96.94**	71.22
**5g**	15.01	-	19.34
**5h**	18.68	26.59	18.49
**5i**	28.63	31.46	19.42
**5j**	20.15	36.24	23.81
**5k**	37.20	51.94	46.36
**5l**	**91.28**	**102.70**	**104.75**
Ascorbic acid	100.00	100.00	100.00
BHT	86.03	92.83	64.87
Trolox	85.87	88.95	56.83

The values obtained for the most active compounds (>90%) are marked in bold.

**Table 3:** Results of the ferrous ions chelation capacity assay.

Compound	Fe^2+^ Chelation Capacity (%)
**3**	-
**5a**	-
**5b**	-
**5c**	-
**5d**	-
**5e**	-
**5f**	12.16
**5g**	-
**5h**	-
**5i**	-
**5j**	10.06
**5k**	-
**5l**	14.58
EDTA	92.78

**Table 4:** The energies of the frontier orbitals HOMO, LUMO, the HOMO−LUMO gap, and the enthalpy of the compounds **5a**–**l**.

Compound	Frontier Orbitals (eV)			Enthalpy (Ha)
	E_HOMO_	E_LUMO_	E_gap_	
**5a**	−5.83	−2.3	3.53	−1691.18
**5b**	−6.24	−1.97	4.27	−1691.19
**5c**	−4.67	−2.21	2.46	−1805.25
**5d**	−6.20	−1.84	4.36	−1691.19
**5e**	−5.84	−1.82	4.02	−1805.68
**5f**	−6.29	−1.93	4.36	−1766.38
**5g**	−6.28	−2.01	4.27	−4264.48
**5h**	−5.76	−1.80	3.96	−1844.96
**5i**	−5.87	−1.75	4.12	−1844.97
**5j**	−5.66	−1.83	3.83	−1920.16
**5k**	−6.01	−1.74	4.27	−1766.40
**5l**	−5.90	−1.89	4.01	−1766.40

**Table 5:** The energies of the frontier orbitals HOMO, LUMO, and the enthalpy of the radicals derived from compounds **5a**–**l**.

Radical of Compound	Position of the Radical	Frontier Orbitals (eV)		Enthalpy (Ha)
		E_HOMO_	E_LUMO_	
**5a**	-	−6.09	−2.44	−1690.54
**5b**	-	−6.68	−2.32	−1690.55
**5c**	-	−6.12	−2.19	−1804.61
**5d**	-	−6.62	−1.94	−1690.56
**5e**	-	−6.13	−2.15	−1805.04
**5f**	*ortho*	−6.34	−1.91	−1765.77
	*meta*	−6.39	−2.23	−1765.77
**5g**	-	−6.63	−2.02	−4263.84
**5h**	-	−6.32	−2.25	−1844.32
**5i**	-	−6.59	−1.92	−1844.33
**5j**	-	−5.78	−2.14	−1919.52
**5k**	*ortho*	−6.54	−1.9	−1765.76
	*para*	−6.42	−2.49	−1765.76
**5l**	*meta*	−6.65	−2.33	−1765.75
	*para*	−6.12	−2.25	−1765.77

**Table 6:** The computed O−H Bond Dissociation Energies (BDE) from the compounds **5a**–**l**.

Compound	Position of the Phenol Group	O−H BDE		
		Hartrees	Kcal/mol	KJ/mol
**5a**	-	0.145	90.826	380.014
**5b**	-	0.145	90.801	379.909
**5c**	-	0.142	88.818	371.613
**5d**	-	0.135	84.431	353.260
**5e**	-	0.142	89.326	373.739
**5f**	*ortho*	0.114	71.561	299.411
	*meta*	0.114	71.668	299.858
**5g**	-	0.146	91.610	383.296
**5h**	-	0.140	87.657	366.755
**5i**	-	0.143	89.627	374.999
**5j**	-	0.141	88.344	369.633
**5k**	*ortho*	0.143	89.684	375.236
	*para*	0.140	87.826	367.464
**5l**	*meta*	0.153	95.971	401.543
	*para*	0.132	82.963	347.117

## Supplementary Materials

The following are available online. Table S1: HOMO and LUMO depicted for the final compounds **5a**–**l**. Table S2: Spin density maps depicted for phenoxyl radicals of the final compounds **5a**–**l**.
