# Supplementary material for: New Phenolic Derivatives of Thiazolidine-2,4-dione with Antioxidant and Antiradical Properties: Synthesis, Characterization, In Vitro Evaluation, and Quantum Studies
Source: Molecules. 2019 May 30;24(11):2060. doi: 10.3390/molecules24112060 (PMC6600258; doi:10.3390/molecules24112060)
Supplement: Supplementary file 1 [file molecules-24-02060-s001.pdf]

Supplementary Materials

New Phenolic Derivatives of Thiazolidine-2,4-dione with Antioxidant and Antiradical Properties: Synthesis, Characterization, In Vitro Evaluation, and Quantum Studies

Gabriel Marc [1], Anca Stana [1,*], Smaranda Dafina Oniga [2,*], Adrian Pîrnău [3], Laurian Vlase [4] and Ovidiu Oniga [1]

1 Department of Pharmaceutical Chemistry, "Iuliu Hațieganu" University of Medicine and Pharmacy, 41 Victor Babeș Street, RO-400012 Cluj-Napoca, Romania; marc.gabriel@umfcluj.ro (G.M.); onigao65@yahoo.com (O.O.)

2 Department of Therapeutic Chemistry, "Iuliu Hațieganu" University of Medicine and Pharmacy, 12 Ion Creangă Street, RO-400010 Cluj-Napoca, Romania

3 National Institute for Research and Development of Isotopic and Molecular Technologies, RO-400293 Cluj-Napoca, Romania; adrian.pirnau@itim-cj.ro

4 Department of Pharmaceutical Technology and Biopharmaceutics, "Iuliu Hațieganu" University of Medicine and Pharmacy, 41 Victor Babeș Street, RO-400012 Cluj-Napoca, Romania; vlaselaur@yahoo.com

* Correspondence: teodora_anca@yahoo.com (A.S.); smaranda.oniga@umfcluj.ro (S.D.O.); Tel.: +40-264-450-529 (A.S.); +40-374-834-851 (S.D.O.)

Table S1. HOMO and LUMO depicted for the final compounds **5a–l**.

Compound	HOMO	LUMO
**5a**	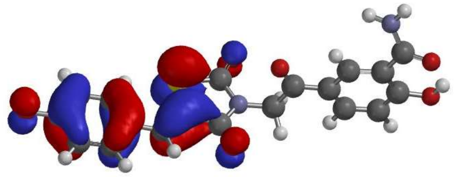	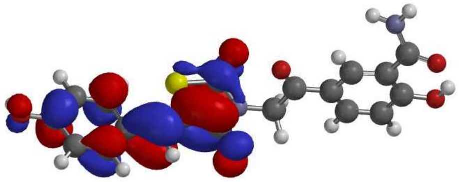
**5b**	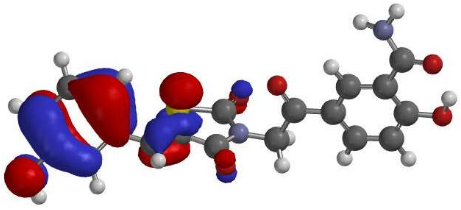	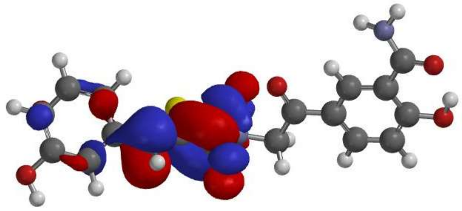
**5c**	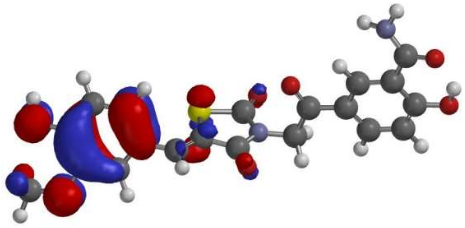	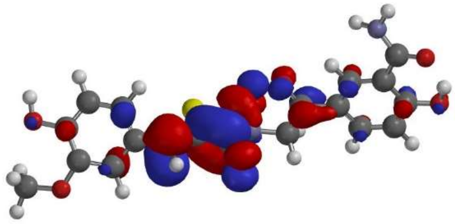

5d

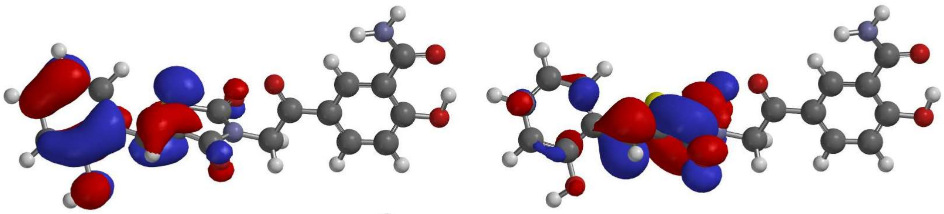

5e

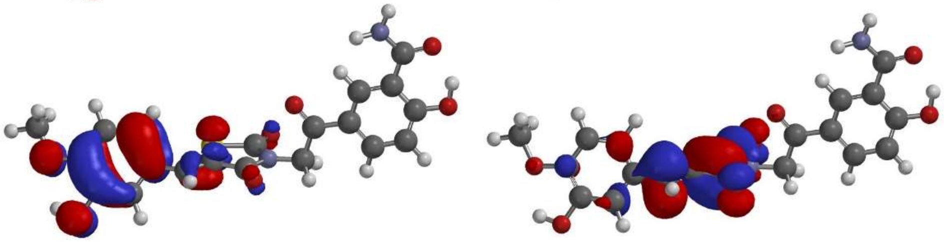

5f

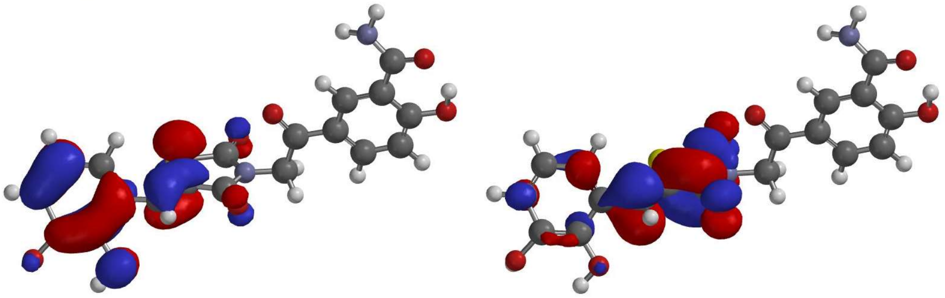

5g

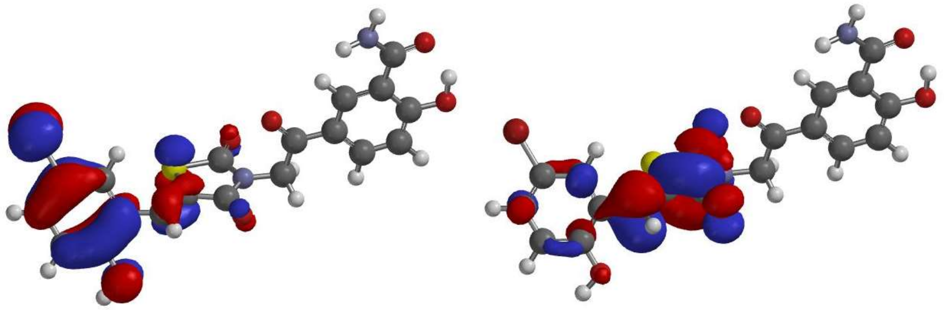

5h

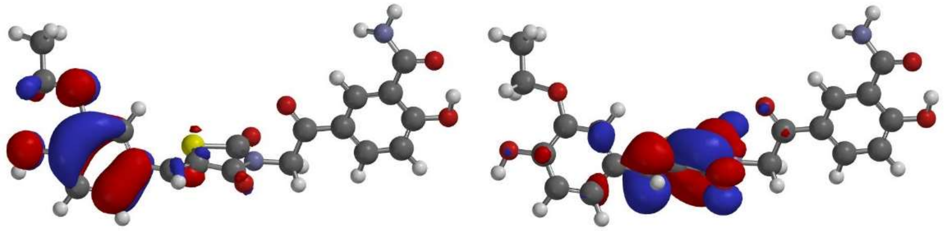

5i

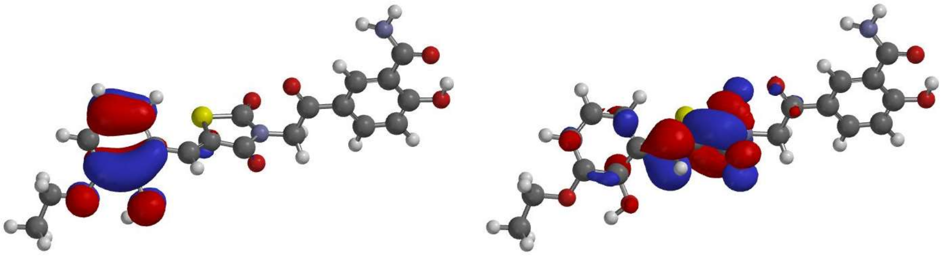

**5j**	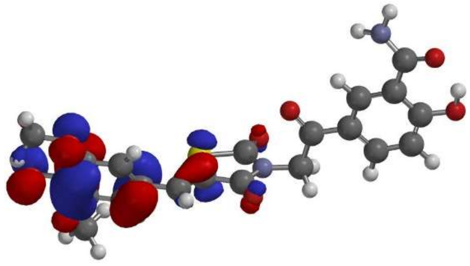	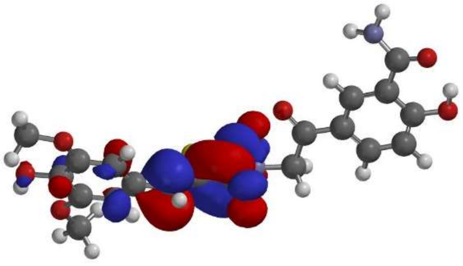
**5k**	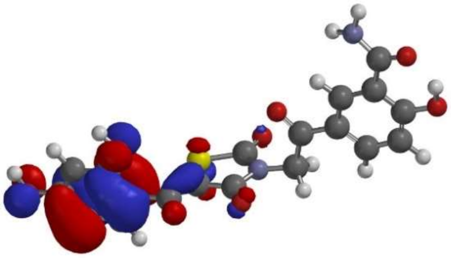	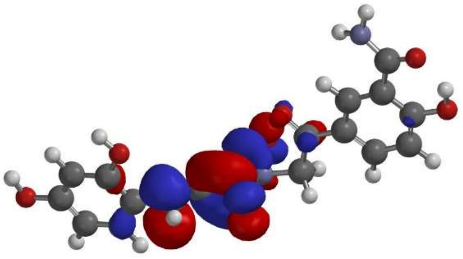
**5l**	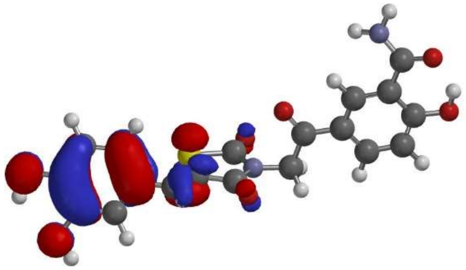	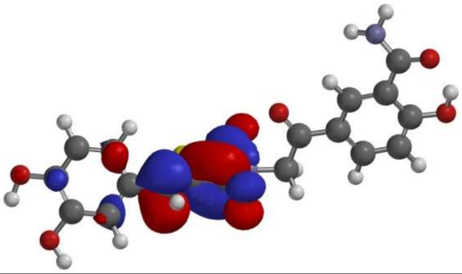

Table S2. Spin density maps depicted for phenoxyl radicals of the final compounds **5a–l**.

Radical	Position	Spin density map
**5a**$^{\cdot}$	-	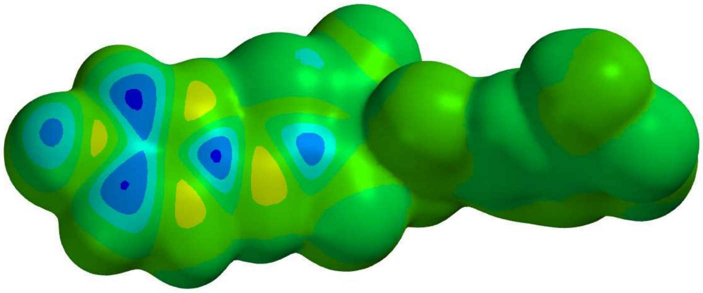
**5b**$^{\cdot}$	-	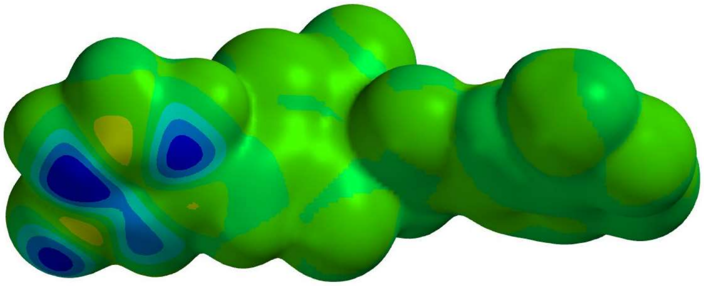

**5c**$^{\cdot}$	-	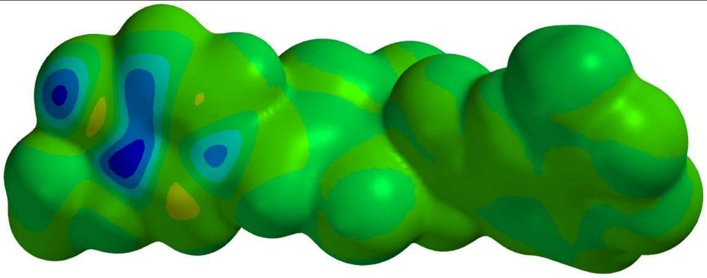
**5d**$^{\cdot}$	-	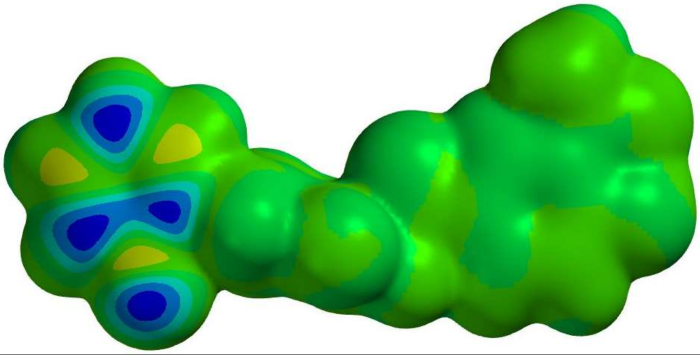
**5e**$^{\cdot}$	-	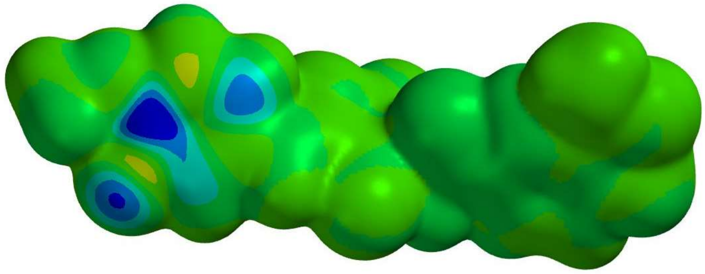
**5f**$^{\cdot}$	*ortho*	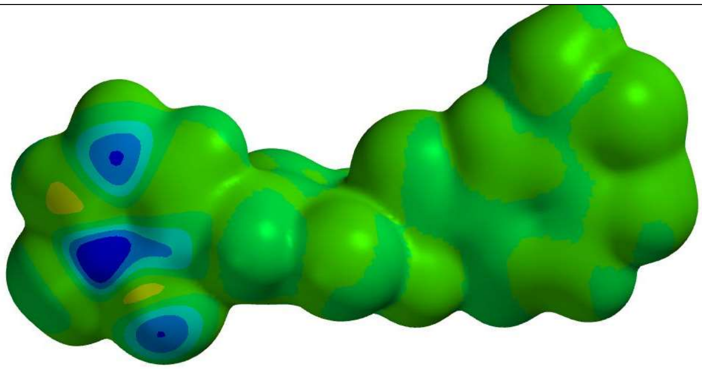
	meta	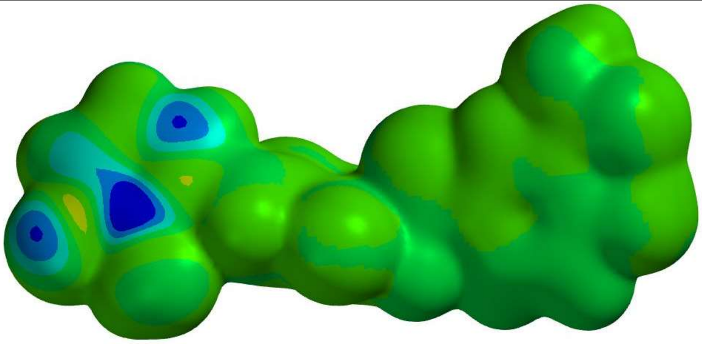

**5g**$^{\cdot}$	-	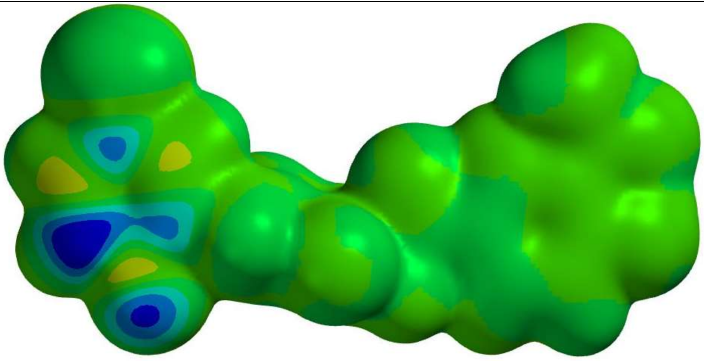
**5h**$^{\cdot}$	-	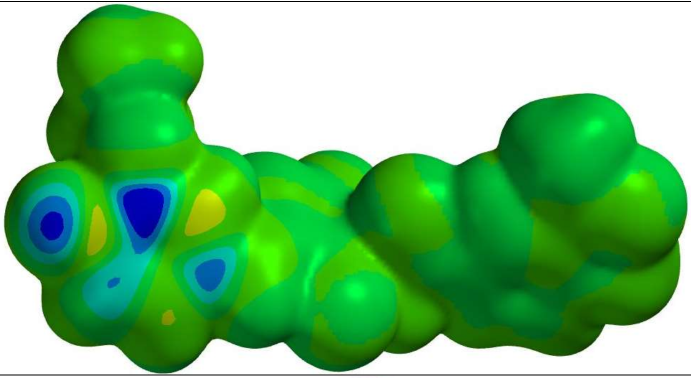
**5i**$^{\cdot}$	-	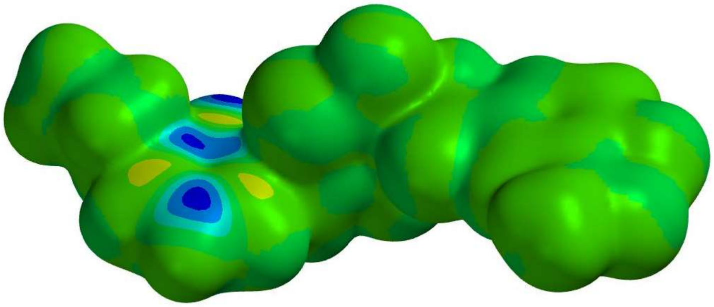
**5j**$^{\cdot}$	-	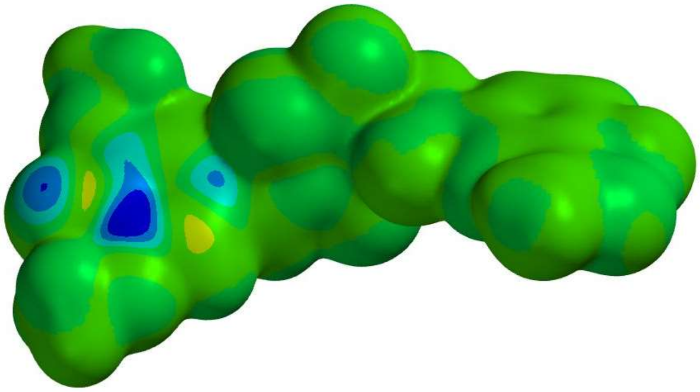
**5k**$^{\cdot}$	*ortho*	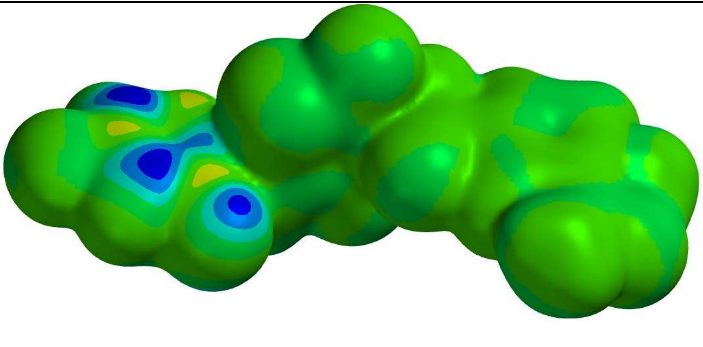

	para	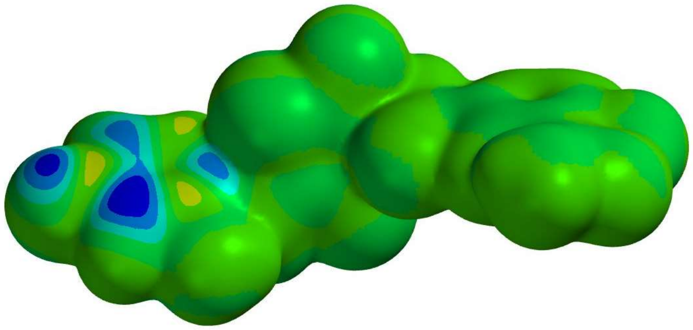
5l$^{\cdot}$	*meta*	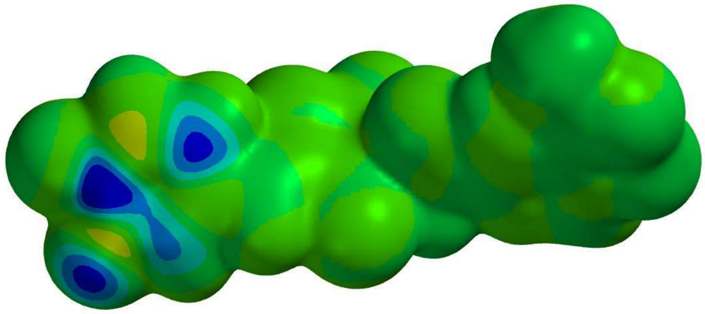
	para	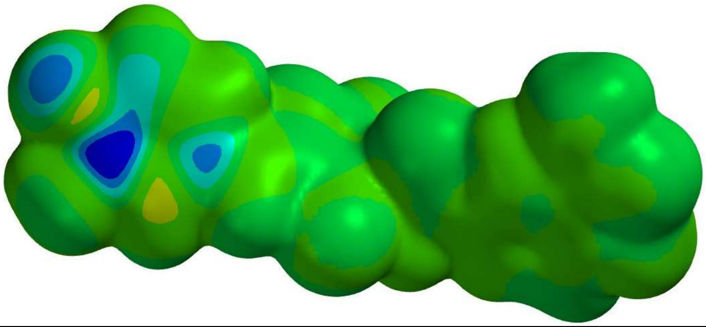
